# Comparing German and Italian food banks: Actors’ knowledge on food insecurity and their perception of the interaction with food bank users

**DOI:** 10.1108/BFJ-11-2017-0626

**Published:** 2018-10-01

**Authors:** Meike Rombach, Vera Bitsch, Eunkyung Kang, Francesco Ricchieri

**Affiliations:** Chair of Economics of Horticulture and Landscaping, Technical University of Munich, Munich, Germany

**Keywords:** In-depth interviews, Poverty, Qualitative content analysis, Food aid, Knowledge forms

## Abstract

**Purpose:**

The purpose of this paper is to investigate food bank actors’ knowledge of food insecurity in Germany and in Italy, as well as interactions between food bank actors and food bank users. The study builds on a knowledge framework from an educational context and applies it to food banks.

**Design/methodology/approach:**

The study uses a qualitative research approach. In all, 22 in-depth interviews were recorded, transcribed and analyzed through inductive qualitative content analysis.

**Findings:**

German and Italian food bank actors interviewed had at least situational knowledge on food insecurity. Some actors of the Italian food bank also showed procedural knowledge. Interactions between food bank personnel and users were affected by feelings of gratitude, shame, anger and disappointment.

**Originality/value:**

The study explores food bank personnel’s knowledge on food insecurity, which appears to be a knowledge gap, even though many prior studies were dedicated to food banks and food insecurity. The study contributes to knowledge systematization to provide best practice recommendations for volunteer-user interaction, and suggests how food bank managers and volunteers’ knowledge can be improved.

## Introduction

Food insecurity is a problem that is occurring in many developed countries, including Western Europe. Approximately 20 percent of all German citizens are affected by the risk of food insecurity and social exclusion (Eurostat, 2015). In Italy, the situation is even worse with approximately 28.7 percent of the population affected by the risk of food insecurity and social exclusion (Eurostat, 2015). In both countries, food insecurity refers to people receiving food aid, and not being able to supply themselves with nutritious, safe and appropriate food to maintain an active life (Riches and Silvasti, 2014, p. 6). People affected are often dependent on social welfare (Tarasuk *et al.*, 2014a), and in addition to their financial struggles, often have psychological and medical problems (Lorenz, 2012a, b; Caraher and Cavicchi, 2014; Lambie-Mumford and Dowler, 2014; Chiu *et al.*, 2016).

Various studies explain how people receiving food aid cope with their situation. These studies emphasize strategies how affected people try to improve their living situation (Lutz, 2011; Pfeiffer *et al.*, 2011; Selke, 2012; Rocha, 2014; Dowler, 2014). Pfeiffer *et al.* (2011) describe solidarity amongst the food poor. Vulnerable groups such as elderly or children are given priority when it comes to food access. As an example, parents tend to restrict their own food supply and ignore their own needs, to make sure their children receive healthy food and their wishes are accommodated (Becker, 2010; Martens, 2011; Pfeiffer *et al.*, 2011; Selke, 2012). Another aspect is the focus on food quantity instead of quality (Pfeiffer *et al.*, 2011; Selke, 2012). Other people use their social networks. Visits to family and friends are not only a social act but also a means to improve the food situation (Pfeiffer *et al.*, 2011). Pfeiffer *et al.* (2011) further states that some people with limited financial resources work illegally or exploit their body to gain income. Overall, food poor people approach their situation in a pragmatic way, even though many food bank users perceive visits to the food bank as shameful (Pfeiffer *et al.*, 2011; Lorenz, 2012a, b; Selke 2012; Riches and Silvasti, 2014; Baglioni *et al.*, 2016).

The German food bank (in German “Tafel”) is a charitable nonprofit organization that redistributes surplus food items to destitute people (Lorenz, 2012a, b). On the national level it is organized as an umbrella association with approximately 900 local food pantries nationwide (Selke, 2011b; Von Normann, 2011; Federal Association of German Food Banks, 2017). Through the food pantries, food donations from agricultural producers and food retailers are distributed to people in need (Von Normann, 2011). In contrast, the Italian food bank (in Italian “Banco Alimentare”) while also collecting surplus food items from agricultural producers and food retailers, acts similar to a wholesaler (Baglioni *et al.*, 2016). These food items are given to different external charitable organization, for instance Caritas (an organization affiliated with the Catholic Church), to distribute them to the needy (Santini and Cavicchi, 2014; Baglioni *et al.*, 2016). The Italian food bank system has been classified by Baglioni *et al.* (2016), building on Tarasuk and Eakin (2005), as a logistic system, because the distribution to food bank users is carried out by an external party. Baglioni *et al.* (2016) classified the German food bank system as a “hybrid system,” because in addition to food the pantries receive directly from donors, the organization’s head office coordinates a distribution center for surplus items within the food pantry network. Still, operations are mainly focused on users, which Baglioni *et al.* (2016) would call a “frontline system.” Since the German food bank has both logistic and frontline features, it is considered a hybrid system.

In both food bank systems, mainly volunteers supervised by administrative staff and food bank managers carry out operations. The volunteers dedicate their time, physical labor and knowledge to the organizations (Federal Association of German Food Banks, 2017; Rete Banco Alimentare, 2017). The knowledge aspect is particularly important as food bank personnel deal with food as a scarce resource and interact with food bank users who are often destitute and ultimately dependent on the food banks. Food insecurity does not only mean insufficient food intake, but is also related to other problems such as financial and psychological difficulties, as well as failure to manage daily routines. Food bank actors’ knowledge about food insecurity is implicitly addressed in several studies but appears to not have been researched directly in published papers. Prior food bank studies in Germany rather focused on sociological, theological and political aspects of poverty (Rohrmann, 2011; Sedelmeier, 2011; Selke and Maar, 2011; Werth, 2011; Lorenz, 2012a, b). Similarly, Italian food bank studies investigated managerial and political aspects of poverty (Garrone *et al.*, 2014; Santini and Cavicchi, 2014; Baglioni *et al.*, 2016). In an effort to fill the knowledge gap described, the present study aims to systematize and compare the knowledge of German and Italian food bank managers and volunteers with respect to food insecurity, and their perception of the interaction with food bank users. The systematization of knowledge allows providing suggestions regarding food bank operations, food insecurity and other related problems as well as training for food bank personnel. Knowing the food bank personnel’s perception of the interaction with users, will also contribute to developing targeted training measures.

## Literature review

In an effort to systematize food bank personnel’s knowledge, an existing knowledge classification system is considered. De Jong and Ferguson-Hessler (1996) presented four forms of knowledge: situational knowledge, conceptual knowledge, procedural knowledge and strategic knowledge. Situational knowledge refers to the knowledge on typical conditions or problem situations in a certain domain. Conceptual knowledge refers to knowing facts, understanding concepts and principles. Procedural knowledge is knowledge that allows identifying actions that can be used to solve problems. Strategic knowledge is the knowledge of what to do and when, by careful planning, informed decision making, as well as effective use of actions (De Jong and Ferguson-Hessler, 1996). These forms of knowledge originate from an educational context. In the present study, they are applied to a context of charitable food assistance.

Several studies addressed the interactions among food bank actors, which provide evidence towards situational and conceptual knowledge about food insecurity. These studies found food bank personnel generally aware that food poor people have financial problems and problems of social inclusion (Lambie-Mumford, 2013; McIntyre *et al.*, 2015). Some food bank actors are aware of food bank users’ problems in other areas of life, because, for instance, the German food bank offers support with authority, medical and bank visits to its users, which may indicate procedural knowledge (Lorenz, 2012a). Further evidence, which relates to procedural and strategic knowledge, addressed collaborations between food banks and governmental agencies and other welfare organization (Lorenz, 2012b; Baglioni *et al.*, 2016; Rombach and Bitsch, 2017). Collaborating welfare organization provides health, education and legal support (Selke, 2011a). In addition, Tarasuk *et al.* (2014b) highlighted food bank networks as an important frame for collaboration, as they allow to exchange of resources among cities and regions. The authors further stated that the scale of operations, fund raising and the control of supply and demand are important competencies that food bank personnel need to possess, when managing food insecurity.

The perception of food bank personnel’s interactions with food bank beneficiaries also is addressed in several studies. These studies show that the interaction between food bank personnel and users is generally positive, but there are incidences of unfriendly treatment, shaming and abuse of food bank users (Pfeiffer *et al.*, 2011; Lambie-Mumford, 2013; Von der Horst *et al.*, 2014; McIntyre *et al.*, 2015). Studies report that asymmetric relationships between volunteers and users lead to tensions and volunteers have usually a different perception of the situation than users. As shown by Lambie-Mumford (2013), volunteers do not necessarily realize when humiliation occurred because the operational business of food banks is busy, or volunteers do not see it as part of their role to be attentive to needs other than food of food bank beneficiaries.

A further mismatch of the situational perception between food bank personnel and food bank beneficiaries is the aspect of expected gratitude for receiving food. Especially, volunteers expect users to show gratitude for their service. If users do not show gratitude, emotionally charged and tense situations occur (Tarasuk and Eakin, 2005; Lorenz 2010a,  b; Becker, 2011; Von Normann, 2011; Selke, 2010, 2011a, b, c; Lambie-Mumford, 2013; McIntyre *et al.*, 2015; Baglioni *et al.*, 2016). Von der Horst *et al.* (2014) pointed out that the issue of gratitude is particular critical when it comes the food items received. They reported an example of misperception between food bank actors and food bank users. The authors explained that food bank volunteers prepared chocolates and candies for seasonal events such as Christmas or Easter. However, user felt humiliated and not taken seriously, because as adults they were more interested in healthy meals.

**Figure 1:**
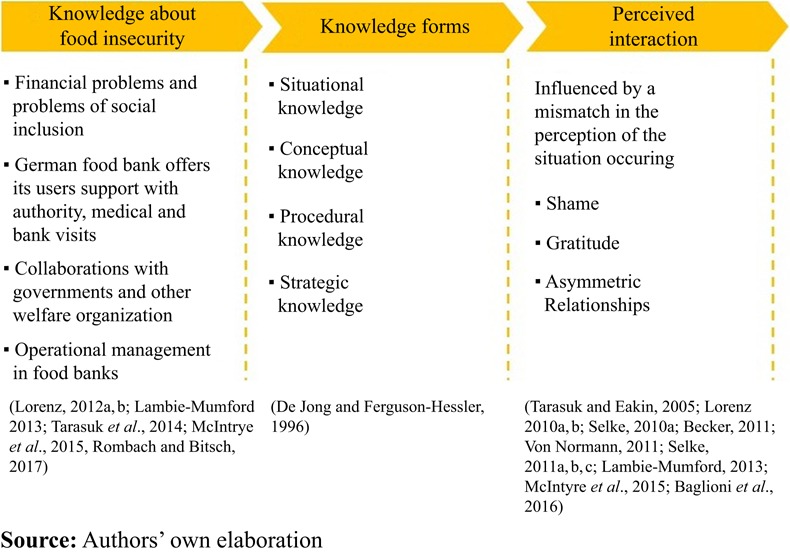
Literature framework of the study

Overall, the analysis of prior studies indicates that food bank actors may have knowledge about food insecurity. However, the extent of knowledge and the specific types of knowledge present remain to be explored. Interactions within the food bank, between food bank actors and food bank beneficiaries, appear to be strongly affected by hierarchical positions, gratitude, shame and a mismatch of perceptions (Figure 1).

## The German and the Italian food banks

The German Food Bank started in 1993 as a social movement, and developed over time into a federal association with an umbrella structure (Federal Association of German Food Banks, 2017). The organization consists of local food pantries, which are united under the federal association. Local pantries differ in size, financial resources, and operational structure (Von Normann, 2011). Within the pantries, food given out to the beneficiaries. In addition, extra services such as providing breakfast to school children, distributing second-hand clothing, and assisting users with medical appointments, banking tasks and dealing with government authorities are offered (Lorenz, 2012a, b; Reiniger, 2011; Von Normann, 2011). Users need to be registered in the German welfare system as financially deprived, and provide official documentation of their need to the food bank, in order to receive these pantries’ services. Among the beneficiaries are 24 percent children and young adults up to 17 years, 65 percent adults, 11 percent are retirees (Von Normann, 2011).

Each beneficiary receives about 3.5 kg of food per week. The value amounts to about eight Euros per capita. The food is either provided free of charge or beneficiaries must pay a symbolic fee. The decision whether to charge or not is made by food pantry managers (Von Normann, 2011). The range of food items includes baked goods, fruits and vegetables and dairy products. Meat, rice and pasta are scarce goods in German Food Banks. Approximately 50,000 volunteers serve at the German Food Bank. Volunteers are required to work 20 hours a month at the food bank and follow the instructions of food bank managers (Von Normann, 2011). Food retail chains, food wholesalers, and food producers are the types of food donors supporting the German Food Bank.

The Italian Food Bank was founded in 1989. The organization also consists of an umbrella organization, uniting 21 regional food bank branches (Banco Alimentare, 2017). These branches collect surplus food items and donations (Garrone *et al.*, 2014). Both, surplus food items and regular marketable food items are redistributed to 8,600 different charitable organizations within Italy serving people in need (Banco Alimentare, 2017). In total, the Italian Food Bank provides food for 1.9 million people (Santini and Cavicchi, 2014). According to Baglioni *et al.* (2016), the Italian Food Bank system, also requires beneficiaries to present proof of neediness and provides extra services that allow food bank beneficiaries to interact with each other. The services are provided by the collaborating charities and usually relate to maintaining contact and social exchange. Given that there are many immigrants without employment, asylum seekers and elderly people with no family among the beneficiaries, opportunities to meet other people are a priority in the Italian system, which is considered equally important as receiving food.

The Italian Food Bank employs 117 staff members, and 1,738 regular volunteers support the organization. At the national food collection day for the poor, the number of volunteers reaches 130,000 (Santini and Cavicchi, 2014). Per year, the organization redistributes 55,800 tons of food (Banco Alimentare, 2017). Similar to the German Food Bank, the Italian Food Bank also collaborates with food retailers, food wholesalers, food producers and catering companies and canteens, providing fresh produce and other groceries (Santini and Cavicchi, 2014).

## Methods

The study uses a qualitative research approach to explore the research question in-depth. A qualitative approach is appropriate when a theory is to be developed, an unknown research topic explored, or a new perspective added to a well-investigated topic (Bitsch, 2005). As the knowledge of food bank actors regarding food waste and food insecurity in Germany and Italy remains largely unknown, a qualitative approach is appropriate. Further, exploring interactions within food banks requires the perspectives of multiple actors involved. Therefore, in-depth interviews were deemed suitable.

Interviews were conducted between March and June 2016. Of the 22 interviewees, five were volunteers at the German food bank, five were food pantry managers at the German food bank, two were volunteers in the Italian food bank, serving in a warehouse, three were managers for the Italian food bank, four were volunteers at Caritas in Italy and three were managers at Caritas (Table I). The first interviewees in each country were recruited through the researchers’ personal networks. Further interviewees were recruited through snowball sampling. This is considered a form of purposeful sampling, where the researchers actively select the most relevant sample to answer the research question (Marshall, 1996; Noy, 2008), which is a common approach in qualitative research. The approach builds on factors that might influence interviewees’ contributions to the research topic and is therefore more relevant than simple demographic stratification (Marshall, 1996). Accordingly, the researchers’ practical knowledge of the research area, and knowledge of literature are important, because these aspects influence the sampling framework (Suri, 2011). To safeguard against potential sampling bias and avoid interruption of the sampling process a multi referral approach was chosen (Heckathorn, 2011). This sampling approach leads to a non-linear sampling pattern and a broader access to interviewees with the goal to avoid sampling from a mostly homogenous network. The first interviewee recruited is asked to provide multiple referrals, especially people with different perspectives. Each new referral is explored and also asked to provide further multiple referrals until saturation is reached.

Each interview lasted 45–90 minutes. The interview guide addressed the following topics, knowledge about food insecurity and food waste, food bank work and structures, as well as interactions between food bank actors and users. The interview guide was tested prior to the field interviews, and during the research process, it was further adjusted. Changes included rephrasing to improve interviewees’ understanding of topics, reordering questions to facilitate the interview flow and adding questions to include issues brought up in prior interviews. This approach is in line with qualitative research procedures (Corbin and Strauss, 1990; Bitsch, 2005).

All interviews were conducted by the second and third author, audio-recorded, transcribed verbatim and analyzed through qualitative content analysis. Each interviewer received interviewer training and advice how to avoid interviewer effects (Bogner and Littig, 2009). During the analysis, the raw text was systematically broken down and constantly revisited by comparing newly encountered information with the data previously analyzed to uncover new themes or improve the understanding of previous ones. The procedure ultimately led to the identification of patterns regarding the actors’ knowledge and interaction patterns in German and Italian food banks.

The most important steps of the qualitative content analysis were coding and the establishment of categories. During open coding, text fragments were labeled and defined. A defined code identifies the main thought behind each text fragment. Categories were comprised of different codes, grouped together according to their meaning. Categories arose through constant revisiting of the transcripts, codes and relationships among codes. Similar to codes, categories were named and defined. In contrast to codes, categories’ definitions consider not only one key thought, but all codes included and their respective definitions. All codes and categories were documented in a codebook.

The analysis was facilitated through the F4 transcription software and the Atlas.ti qualitative analysis software. The transcription was a simple script, where dialect and colloquial language were adjusted to standard language. Following Halcomb and Davidson (2006) nonverbal language and irrelevant content were omitted. The transcription followed the rules suggested by Dresing *et al.* (2015). The Atlas.ti software is dedicated to coding and establishment of categories. It allows revisiting the text in a systematic manner and includes features for annotation and coding, as well as memo writing. Memo writing refers to the researchers’ documentation of ideas on coding and the analysis process. It is the first step towards theory building and is typically practiced throughout the entire process of analysis. Since memos are analytical and conceptual notes, they serve to clarify the researchers’ thoughts and improve the exchange of ideas among the research team. Also, memos contribute to structure and abstraction of data (Birks *et al.*, 2008; Charmaz, 2015).

## Results and discussion

German and Italian food bank personal shared their knowledge about food insecurity and food bank interactions in their respective countries. Managers and volunteers showed at least situational knowledge in both countries. Furthermore, managers and volunteers shared experiences and examples from their social engagement. When sharing their perception of their interaction with food bank beneficiaries, German as well as Italian managers and volunteers emphasized the beneficiaries’ positive or negative emotional reactions when receiving food. Particularly Italian food bank personnel described a variety of problems in the context of food insecurity.

Volunteers at German food pantries defined food insecurity as referring to the unsatisfied basic human need for food. Volunteers did not identify causes, but talked about the condition itself. The volunteers reported that even though they know that food insecurity exists in Germany, they have difficulties to relate to it. Some stated that they face this difficulty, because they have never experienced hunger in a physical sense, only in a psychological sense. Other volunteers wondered about the fact that in a relatively wealthy country like Germany, there are citizens that do not have enough food:

I guess food security is the big basic of a human being, basically to have enough food to function. I think it is astonishing, you always think Germany is a big and rich country but there are still people who need help to have like the basic of basics. I do not know […] I never had to go to bed when I am hungry. The food bank is an organization that helps people to have food security.

(Volunteer at the German food bank, female, 20–30 years old)

Exactly, so now they [food bank users] are very dependent on the food bank because they have no income. They are unemployed or their retirement benefits are very small, close to subsistence level. That is why they are dependent in food donations.

(Manager at the German food bank, male, 50–60 years old)

Volunteers raised the question whether this is possible, given the sophisticated welfare system in Germany. Food pantry managers brought up similar thoughts. Managers correctly identified that food insecurity exists in Germany. They related the cause of food insecurity mainly to the lack of income. They emphasized that people could be food poor when they receive public assistance, when they are long-term unemployed, receive only small retirement benefits or are refugees in Germany. Both groups, volunteers and managers, stated that they strongly believe that people are able to supply themselves with food and are not at risk of starvation in Germany even without of the food bank due to the German welfare system.

This result confirms prior studies that found some food bank users use food pantry services, because they want to save money for other purposes (Selke, 2011a, b; Lorenz, 2012b). Further, it is noteworthy that even though prior studies mention recent cuts to the German welfare system (Von Normann, 2011), interviewees in the present study do not consider these cuts as particularly problematic. Both groups, managers and volunteers shared mainly superficial situational knowledge. However, they may know more than they articulated.

In contrast, Italian volunteers directly related food insecurity to shame, social stigma, and poverty. The volunteers discussed differences between regions, especially comparing southern Italy (poorer region) and northern Italy (wealthier region), in terms of poverty and food insecurity. Volunteers reported that many people using food pantries are unemployed or belong to families where food insecurity and lack of education have been present for generations:

There are poor people that come from poor families who come from poor families. Poverty is in their blood. It seems to be a state that they want to maintain. If one of the children of these parents do not think so, s/he needs to leave the family. As long as s/he stays there, s/he cannot leave poverty behind. It is strange, some families seem to feel good in these conditions. This is very serious. If they pass on this mindset to their children, poverty lasts longer.

(Volunteer at the Italian Caritas, male, 50–60 years old)

Italian volunteers show more situational knowledge than volunteers in Germany, given that they are able to identify geographical differences, unemployment, and education as causes and identify a social component, such as stigma. Stigmatization was also emphasized in prior studies (Tarasuk and Eakin, 2003; McIntyre *et al.*, 2015).

Italian food bank managers have comprehensive situational knowledge. They addressed shortcomings in the Italian welfare system as a cause of increasing dependency on food aid. Managers described food insecurity as the top of the “poverty iceberg.” Similar to volunteers, they saw unemployment and lack of education as the main reasons for food insecurity and poverty. They further identified children and elderly people as vulnerable groups who are often food poor:

If you have a family and want to support them, you need to bring money home. The work is what gives you dignity. I think work is the basis of everything. Here are many people, even women, including foreign women, ready to do any kind of job. In short, they have realized a job and an education is the only way out of this condition and to help their family.

(Manager at the Italian Caritas, female, 50–60 years old)

I realized I cannot fully respond to their [food bank users] needs, also because the needs they have is not only food. Behind there, there are many other things. Let us say, I cannot satisfy all their demands they have towards life.

(Manager at the Italian Caritas, male, 50–60 years old)

Managers also shared procedural knowledge. They explained that the Italian food bank, Caritas and other organizations have created an assistance network that focuses on helping destitute people with various problems in their life. Managers further addressed that the attempt to end food insecurity with surplus food items has only short-term effects. They consider it as temporary assistance but not as a solution, because food quantities are not sufficient. Overall, they consider food insecurity as a form of poverty, and as a cultural, as well as an educational problem. The findings correspond partially with Tarasuk *et al.* (2014b) who emphasized the importance of supply and demand control in food banks, as the number of beneficiaries outweighs the available quantities food items. The identification of assistance networks can be seen as an extension of earlier studies identifying individual collaborations between food banks and other welfare organizations (Lorenz, 2012b; Baglioni *et al.*, 2016; Rombach and Bitsch, 2017).

Comparing the knowledge of interviewed German and Italian food bank volunteers and managers, managers were found to have more profound knowledge than volunteers. The difference in knowledge is likely due to the fact that managerial duties require knowledge in multiple areas, whereas volunteers usually focus on the specific tasks assigned.

Regarding cultural differences, the Italian interviewees appear to have deeper knowledge, in particular managers who not only showed situational but also procedural knowledge. Differences in knowledge between interviewees cannot be explained by the lack of education of interviewees or by organizational differences, even when considering the different organizational setup of food banks in both countries. Most likely, the differences in the economic situation and in the welfare systems explain the knowledge difference. It is somewhat surprising that only situational and procedural knowledge was shared by interviewees, as food bank managers were expected to have strategic knowledge due to the variety of operations their positions require them to manage. Specifically, internal business information was not shared, which could indicate that the interview situation was not conducive to sharing this kind of information and other forms of knowledge. As expected, conceptual knowledge was not found, because conceptual knowledge is more relevant in an educational context than in the context of the present study.

Regarding how they perceive interactions with food bank beneficiaries, food bank managers and volunteers in both countries reported that in the majority of cases interactions were positive. Interviewees highlighted that users typically like to come and thank them for the food and their commitment. However, there were also cases when users did not show gratitude which is upsetting for the volunteers serving them. The mixture of feelings, such as shame, gratitude, anger and disappointment is causing tensions in interactions in both countries (Table II). These findings confirm prior studies (Tarasuk and Eakin, 2005; Selke, 2011a, b; Lorenz, 2012b; Lambie-Mumford, 2013; Van der Horst *et al.*, 2014; McIntyre *et al.*, 2015).

The main difference between serving destitute people in Italian and German food banks is that, in negative interactions, German users were described as sharing negative feelings more subtly than Italian users who were more direct. Italian managers shared cases when dissatisfied users blamed the Italian food bank or Caritas in online forums for providing poor service and food products. These differences are likely cultural, but they could also be related to the specific people involved. In terms of how food bank personnel perceived interactions with users, both food banks showed the same problems; but user response appears different. The difference in user response maybe be dependent on the availability of other sources of assistance for the users. Most likely, if users are very dependent, as seems to be the case in Italy, reactions are more emotionally charged. Given that neither German nor Italian managers reported negative consequences for users who were perceived as behaving inappropriately in interactions, it can be assumed that a mixture of individual perception and knowledge on food insecurity moderates the food bank personnel’s reactions in those situations.

## Conclusions

The study investigated food bank actors’ knowledge of food insecurity in Germany and Italy, as well as their perception of the interactions between food bank actors and food bank users. Results showed that all food bank actors interviewed had at least situational knowledge of food insecurity. Managers from the Italian food bank also showed procedural knowledge. Interactions between food bank personnel and users were affected by a mismatch of perception within the situation and feelings of gratitude, shame, anger and disappointment.

As the behavior of food bank users, for instance not showing gratitude is perceived as inappropriate by volunteers, it shows that food bank personnel is not able to fully recall their situational knowledge about food insecurity at all times, in particular in tense situations. Therefore psychological training and role playing exercises designed to teach food bank personnel how to reflect and act appropriately if users are angry, sad, or do not show gratitude could be beneficial. Kinnane *et al.* (2011) who researched the effects of volunteer education and training, suggested that role-plays are helpful in volunteer training, because they exercise a realistic situation and provide confidence to the volunteers. Particularly for food bank volunteers this type of training also would lead to improved interaction skills. In an effort to increase knowledge, in the German case, volunteers should be provided with the opportunity to serve in different areas within the food bank. This would help them to get a more comprehensive picture of resources, structures, partners, networks, logistics and other operations. In the Italian case, such training could be achieved through excursions and visiting the partner organizations. In both cases, conceptional knowledge can be provided in lecture formats, because more knowledge related to food insecurity and related problems, as well as regarding food bank systems will complement the practical volunteer work.

As a potential measure for trained food bank volunteers who still struggle with relating to the various issues of food insecurity could include learning about the perspective of a needy person from the inside. Volunteers could be asked to live for two or three weeks on a budget similar to that obtained from the German respectively the Italian social welfare system to experience these conditions. As the experience is limited to a short duration, and not a permanent condition, volunteers may not experience the extreme struggles described by Pfeiffer *et al.* (2011). Yet, the tight budget conditions should allow the volunteers to better relate to the users’ situation with respect to financial and social struggles.

Additional training in strategic management could potentially help food bank managers, complementing their procedural knowledge. One important aspect is the control of supply and demand, as in both countries food demand outweighs available quantities of food items. Managers should seek joint solutions together with governmental authorities. In the German case, refugees recently were banned from certain food pantries as a solution to the problem of growing demand and stagnation or shrinking supply. As this approach garnered wide attention in the media (see, e.g. Washington Post, 2018) and damages the food bank’s reputation, addressing the supply side would be a more suitable approach.

Future research could explore the effects of structures and size on knowledge and competencies of food bank personnel. Furthermore, research on human resource management practices in food banks could shed light on conflicts among food bank beneficiaries and food bank personnel. Interaction problems as well as conflicts not likely to be cause by knowledge deficits alone, but by a multitude of other factors, including structural and managerial factors.

## Figures and Tables

**Table I:** Interviewees and their background

Country	Position and organization	Duty	Gender
Italy	Volunteer at Caritas	Supervision of refugees	Female
Italy	Volunteer at Caritas	Sorts and inspects food	Female
Italy	Volunteer at Caritas	Prepares food	Male
Italy	Volunteer at Caritas	Prepares food	Female
Italy	Volunteer at Caritas	Packs food	Female
Italy	Volunteer at Caritas	Serves beneficiaries	Female
Italy	Pantry manager at Caritas	Managerial and administrative duties	Male
Italy	Panty manager at Caritas	Managerial and administrative duties	Male
Italy	Pantry manager at Caritas	Managerial and administrative duties	Male
Italy	Volunteer at the Fondazione banco Alimentare	Administrative duties	Female
Italy	Volunteer at the Fondazione banco Alimentare	Sorts and inspects food in the warehouse	Male
Italy	Volunteer at the Fondazione banco Alimentare	Coordinates warehouse logistics	Male
Italy	Manager at the Fondazione banco Alimentare	Managerial and administrative duties	Male
Germany	Volunteer at a local food pantry of the German food bank	Sorts and inspects food	Female
Germany	Volunteer at a local food pantry of the German food bank	Serves beneficiaries	Female
Germany	Volunteer at a local food pantry of the German food bank	Serves beneficiaries	Female
Germany	Volunteer at a local food pantry of the German food bank	Drives food truck	Male
Germany	Pantry manager at a local food pantry of the German food bank	Managerial and administrative duties	Male
Germany	Pantry manager at a local food pantry of the German food bank	Managerial and administrative duties	Female
Germany	Pantry manager at a local food pantry of the German food bank	Managerial and administrative duties	Male
Germany	Pantry manager at a local food pantry of the German food bank; co-founders of the German Food Bank	Managerial and administrative duties	Female
Germany	Manager at a local food pantry of the German food bank and state representative	Managerial and administrative duties	Male

**Table II:** Codes for the category “Factors leading to emotionally charged interactions in food banks” with interview excerpts

Code	Excerpt
Shame Visiting the food bank is a shameful experience for food bank users, because the visit at the food bank makes them aware that they are partly socially excluded	“[…] It is a psychological moment. They should not feel shame. They should not have the feeling that what they get is not valuable, and it is just food that others do not want to eat any longer. There would be no dignity” (Manager at the German food bank, female, 70–80 years old) “You always need to take into account that there are also people who feel ashamed. I need to admit there are a few families who ask me to prepare a food bag and ask me if I could bring it to them. I do not say to their home, but to an agreed place, because they are ashamed to come here” (Vice president at the Italian Caritas, male, 50–60 years old)
Gratitude Food bank volunteers expect food bank users to show gratitude for their service	“They [food bank users] have hardly anything left to live. We often hear from our guests, if we would not exist, they would not know how to make ends meet […]” (Volunteer at the German food bank, female, 60–70 years old) “Some are happy. […] Indeed, before they leave they come to the kitchen and thank us. They are so glad for what we have prepared” (Volunteer at the Italian Caritas, female, 40–50 years old)
Need Food bank volunteers have a particular understanding of need, and be deserving to receive food aid. They expect users to behave aligned to this understanding, e.g. come regularly. If users do not show this behavior they are punished or blamed	“If they are not coming without an excuse more than three times, they are dropped from the list and then a new one is coming. We suppose they are not in need” (Volunteer at the German food bank, female, 60–70 years old) “It is the crumbs that matter, right? We need to recover the maximum quantities of food waste, to feed the people who have nothing. If you are in need, then even the crumbs can count” (Volunteer at the Italian food bank, female, 20–30 years old)
Dissatisfaction Food bank beneficiaries show different forms of negative reactions concerning their food bank experiences and interactions at the food bank	“There are a lot of people coming, and we have a limited number of people here. If we offer the products and someone just says ‘yeah’ [expression of dissatisfaction with product, but does not want to refuse], some team members do not like it” (Volunteer at the German food bank, male, 60–70 years old) “A lady loaded up pictures on Facebook of the food we gave her and said this is the crap that they gave me at Caritas today” (Manager at the Italian Caritas, male, 50–60 years old)
